# Stereotactic radiotherapy for spinal and non-spinal bone metastases: a patterns-of-care analysis in German-speaking countries as part of a project of the interdisciplinary Radiosurgery and Stereotactic Radiotherapy Working Group of the DEGRO/DGMP

**DOI:** 10.1007/s00066-025-02387-y

**Published:** 2025-03-18

**Authors:** F. Nägler, N. Gleim, I. Seiler, A. Rühle, K. Hering, C. Seidel, E. Gkika, D. Krug, O. Blanck, C. Moustakis, T. B. Brunner, A. Wittig-Sauerwein, N. H. Nicolay

**Affiliations:** 1 Department of Radiotherapy and Radiation Oncology, University Hospital Leipzig, Stephanstraße 9a, Leipzig, Germany; 2 Department of Radiotherapy and Radiation Oncology, University Hospital Bonn, Venusberg-Campus 1, Bonn, Germany; 3 Department of Radiotherapy and Radiation Oncology, University Medical Center Hamburg-Eppendorf, Martinistr. 52, Hamburg, Germany; 4 Department of Radiation Oncology, University Medical Center Schleswig-Holstein, Campus Kiel, Arnold-Heller-Straße 3, Haus L, Kiel, Germany; 5 Department of Radiation Oncology, Medical University of Graz, Auenbruggergasse 32, Graz, Austria; 6 Department of Radiation Oncology, University Hospital Würzburg, Josef-Schneider-Str. 11, Würzburg, Germany; 7 Comprehensive Cancer Center Central Germany, Partner Site Leipzig, Leipzig, Germany

**Keywords:** Bone metastases, Palliative treatment, Stereotactic radiotherapy, Local ablative treatment, Patterns of care

## Abstract

**Background and purpose:**

Bone metastases constitute a common indication for both conventional radiotherapy (RT) and stereotactic body radiotherapy (SBRT). Although in recent years guidelines have been proposed for SBRT of spinal and non-spinal metastases, little is known about the use of bone SBRT and the actual patterns of care in German-speaking countries.

**Material and methods:**

We performed an online survey among radiation oncologists (ROs) registered with the interdisciplinary Radiosurgery and Stereotactic Radiotherapy Working Group of the German Society of Radiation Oncology (DEGRO) and the German Society for Medical Physics (DGMP) to collect valuable and robust cross-sectional data on patterns of care for bone SBRT in German-speaking countries.

**Results:**

Of the registered ROs, 35.5% (75/211) completed the online survey. ROs working in high-volume centers irradiating more than 100 patients with bone metastases annually represented the largest group, with 58.7%. Ablative SBRT was mostly performed for bone oligometastases (78.7%). For symptom-directed palliative radiotherapy, the majority of responding physicians (84.3%) still mostly recommend moderately hypofractionated treatment. Nevertheless, 60.9% of participating ROs stated using bone SBRT at least occasionally, also for palliative purposes such as pain control. Our survey also revealed a certain reluctance for the concomitant use of systemic therapies with bone SBRT and heterogeneity regarding target volume definition and dosing for bone SBRT.

**Conclusion:**

Our survey demonstrates that bone SBRT for spinal and non-spinal metastases for oligometastatic disease (OMD) is broadly available in clinical routine care in German-speaking countries. A large heterogeneity regarding indications, dose, and fractionation concepts remains, requiring further efforts for standardization of bone SBRT.

**Supplementary Information:**

The online version of this article (10.1007/s00066-025-02387-y) contains supplementary material, which is available to authorized users.

## Introduction

Bone metastases (BoM) constitute a common problem for many patients with solid tumors, especially for those with lung, breast, and prostate cancers [1, 2]. Depending on their size and localization, BoM are associated with a high risk of skeletal-related events (SRE) such as pain, spinal cord compression, or pathologic fractures, which, in turn, often require hospitalization and urgent intervention. Approximately one third of all BoM are considered complicated lesions, mostly defined by the appearance of pathologic fracture (42.1%) and neurologic compromise (36.3%) [3]. The standard of care for symptomatic BoM is palliative external-beam radiotherapy (RT), providing successful pain relief with minimal toxicity [4, 5]. For uncomplicated BoM, overall pain response rates of about 60% and complete pain response rates of 23% for single-fraction RT and 24% for multiple-fraction RT have recently been reported in a large meta-analysis [6]. As a result of improved diagnostic options and increasing long-term survival owing to new systemic therapies, the role of available high-dose ablative RT is becoming increasingly crucial, as it may improve long-term tumor and symptom control, especially in the context of oligometastatic disease (OMD).

The concept of OMD was introduced by Hellman and Weichselbaum, defining an intermediate state between localized and systemic disease, where radical treatment to all sites could result in long-term survival [7]. Recent consensus and guideline publications by the European Society for Radiotherapy and Oncology (ESTRO) and the European Organisation for Research and Treatment of Cancer (EORTC) proposed a subdivision of OMD into de-novo OMD, repeat OMD, and induced OMD based on the heterogeneity often seen in clinical routine [4, 8].

In recent years, stereotactic ablative body radiotherapy (SBRT) has found its way into treatment guidelines for various malignant and benign tumors [9, 10]. In the context of OMD, SBRT might provide long-term freedom from progression or even cure in highly selected patients in the case of total metastatic ablation, and some trials have shown a benefit in progression-free survival and overall survival especially for limited BoM [11–13]; however, subsequent larger randomized trials have failed to consistently replicate these survival advantages across different patient populations, especially for breast cancer [14–16]. While guidelines for SBRT for spinal and non-spinal metastases have been proposed, important questions regarding the optimal choice of locally ablative vs. palliative bone SBRT, optimal dose schemes, and patient selection for SBRT remain [17–23]. Moreover, little is known about the use of bone SBRT and the actual patterns of care pertaining to both palliative treatment and locally ablative therapies for bone oligometastases in German-speaking countries. A recent online survey in Germany on conventional and stereotactic RT for spinal metastases showed that SBRT was not comprehensively practiced, and the choice of dose regimen and target volume definition varied considerably between centers [24]. In order to more clearly define the patterns of care and treatment concepts for bone SBRT offered in Germany, Austria, and Switzerland, we performed an online survey among all medical members of the interdisciplinary Radiosurgery and Stereotactic Radiotherapy Working Group (DEGRO-WG RS and SRT) of the German Society of Radiation Oncology and the German Society for Medical Physics (DGMP). The aim of this survey was to outline the treatment routine for bone SBRT in terms of patient selection, dose and target volume concepts, and the role of SBRT within the oncologic treatment algorithm.

## Materials and methods

We created an online survey consisting of 28 items. Questions concerned the characteristics of treatment centers and expert radiation oncologists (ROs); institutional treatment standards and follow-up procedures for BoM; indications for bone SBRT; and details of treatment planning, preparation, and application. The survey was an official project of the DEGRO-WG RS and SRT; it was set up using SurveyMonkey® (SurveyMonkey Inc., San Mateo, CA, USA) and was distributed among all members of the DEGRO-WG RS and SRT with a specific invitation to registered ROs on April 24, 2024. Two reminders were sent 14 days apart. Data collection was closed on June 30, 2024.

Data were collected centrally and analyzed with R Statistical Software version 4.3.1 (R core team, open source) [25]. Descriptive statistics were used to quantify all answers, χ^2^ tests or Fisher’s exact tests were used for subgroup analyses. Test results with *p* < 0.05 were considered statistically significant. The entire survey with all answers is available in Supplementary Table 1.

## Results

### Participants and participating centers

The survey was circulated among all 211 registered medical members of the DEGRO-WG RS and SRT in Germany, Austria, and Switzerland. Of the registered radiation oncologists, 35.5% (75/211) completed the online survey, 46.7% (35/75) of whom were working in university hospitals and 26.7% (20/75) each in non-university hospitals and outpatient radiotherapy facilities. Most responders were board-certified radiation oncologists (68/71), almost three quarters (74.7%; 53/71) of them working in a leading position (senior consultant physicians: 35/71; 49.3%; departmental heads: 18/71; 25.4%) at their respective institution (Supplementary Fig. 1). Centers treating more than 100 patients with BoM with radiotherapy annually represented the largest group, with 58.7% of participants (44/75), followed by centers with 51–100 patients per year (33.3%; 25/75). The majority of radiation oncologists (56%; 42/75) reported treating about 10–30% of these patients with stereotactic radiotherapy (Supplementary Fig. 2). Comparing university medical centers and non-university centers, there were significant differences in the number of patients treated for BoM annually: non-university centers predominantly reported treating between 51 and 100 patients per year, while university centers treated more than 100 patients with BoM annually (*p* < 0.05). There were no significant differences in the percentage of patients with BoM treated with SBRT, but the rate of bone SBRT tended to be slightly higher in university centers. As an example, 68.6% (24/35) reported treating 10–30% of these patients with SBRT vs. 45% (18/40) in non-university centers (Supplementary Fig. 3).

### Treatment indications for palliative and stereotactic radiotherapy of bone metastases

**Fig. 1:**
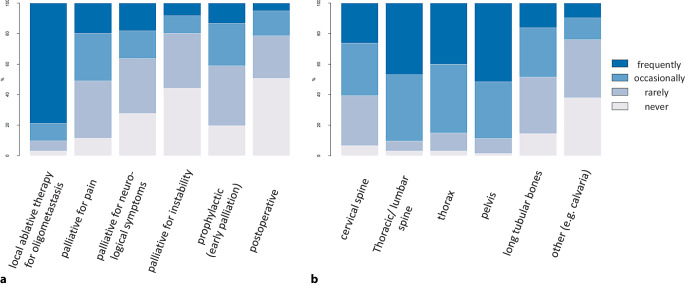
Treatment indications for stereotactic body radiotherapy (SBRT) of bone metastases (**a**) and localizations amenable to bone SBRT in participating centers (**b**)

For palliative irradiation of BoM, the majority of responding physicians (84.3%; 54/64) recommended moderately hypofractionated treatment concepts (single doses of 2.5 to 3.5 Gy); 60.9% of ROs (39/64) reported using stereotactic ablative concepts for palliation of BoM at least occasionally. However, 50.8% (31/61), 44.3% (27/61), and 27.9% (17/61) of answering physicians regarded postoperative bone radiation, bone instability, and new or progressing neurological deficits, respectively, as contraindications to bone SBRT (Fig. 1). Of the included ROs, 57.4% (35/61) performed stereotactic ablative treatment of BoM most frequently in the context of OMD. In this context, 41.3% of participants (26/63) defined osseous OMD as ≤ 5 BoM; 30.2% of respondents (19/63) answered that they deviate in their definition of oligometastases depending on clinical, anatomic, and histologic factors.

Almost two thirds of ROs (60.7%; 37/61) stated that they decide for or against bone SBRT regardless of histology. However, small cell lung cancer was the most frequently mentioned histology for which no bone SBRT was recommended (31.4%; 16/51), followed by disseminated hematological malignancies (27.5%; 14/51) and multiple myeloma (21.6%; 11/51).

The pelvic bones were reported as the most common site of bone SBRT (frequently: 51.6%; 32/62; occasionally: 37.1%; 23/62), followed by the thoracic/lumbar spine (frequently: 46.7%; 29/62; occasionally: 43.5%; 27/62) and the ribs, scapula, or sternal bone (frequently: 40%; 24/60; occasionally: 45%; 27/60). SBRT for BoM of the cervical spine was reported to be applied occasionally by 34.4% (21/61) and frequently by 26.2% (16/61), while bone SBRT for the long bones of the extremities was used occasionally by 32.3% (20/62) and frequently by 16.1% of participants (10/62; Fig. 1).

**Fig. 2:**
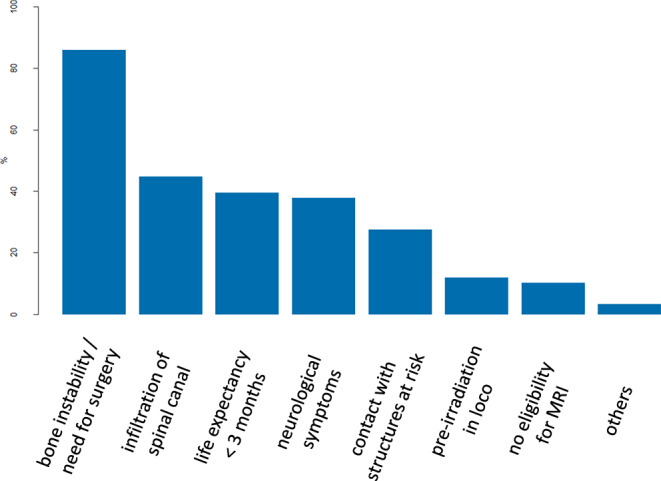
Absolute contraindications to bone stereotactic body radiotherapy as mentioned by the survey participants

Bone instability with the need for surgical stabilization was considered as the most common absolute contraindication to SBRT of BoM (86.2%; 50/58), followed by relevant spinal cord compression (44.8%; 26/58) and limited life expectancy of less than 3 months (39.7%; 23/58; Fig. 2).

### Patterns of care for combining bone SBRT with systemic therapies

Only 4.9% (3/62) of participating ROs declined any treatment combination of bone SBRT with systemic therapies. However, 30.6% (19/62) of ROs reported concomitant use of systemic therapies, and 32.3% (20/62) felt safe combining bone SBRT with sequential systemic treatments. Of all respondents, 32.3% (20/62) had no concerns about combining SBRT with both sequential and concomitant systemic therapy.

**Fig. 3:**
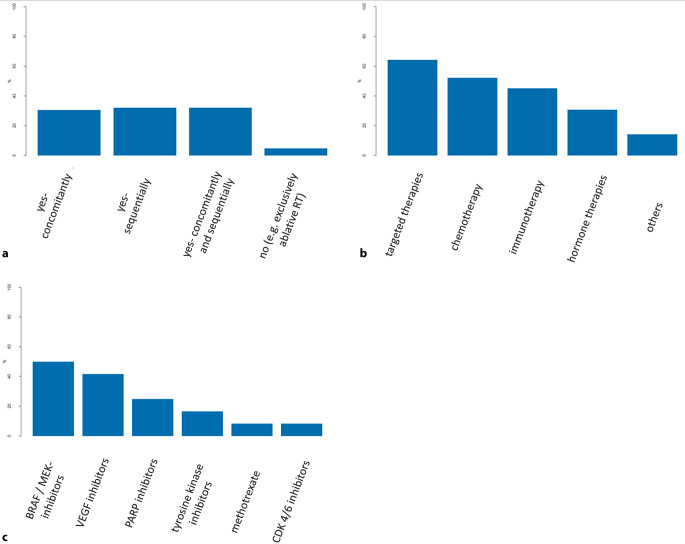
Patterns of care for combining bone stereotactic body radiotherapy (SBRT) with systemic therapies. **a** Continuation of treatment with systemic therapies in combination with bone SBRT; **b** types of systemic therapy for which no concomitant use with SBRT is recommended; **c** substance classes for which no concomitant use with SBRT is recommended

Concerning specific systemic therapies, many of the participating physicians declined concomitant use of either targeted therapies (27/42; 64%), chemotherapy (22/42; 52.4%), or immunotherapy (19/42; 45.2%) with bone SBRT. Especially the combination of BRAF/MEK inhibitors with bone SBRT was negated most frequently (6/12; 50%). Interestingly, 30.9% (13/42) of ROs expressing concerns about systemic therapies reported stopping hormone-modifying therapy such as androgen deprivation therapy during bone SBRT (Fig. 3).

Adding bone-modifying agents to bone SBRT was recommended by 56.5% of ROs (35/62) for most treated patients (i.e., > 50% of patients) and by 35.5% (22/62) for at least some patients (25–50% of patients).

### Treatment planning and application of bone SBRT

Reproducible patient positioning for bone SBRT was seen as a key issue by treating ROs, and the majority of participants reported regular use of dedicated positioning equipment such as rigid masks and/or vacuum cushions (79.6%; 39/49). Additional motion-management systems to control patient surface areas or respiratory motion are used by 55.1% of treating ROs (27/49).

For treatment planning, the vast majority of physicians recommended additional diagnostic imaging such as Magnetic resonance tomography (MRI) (91.9%; 45/49), diagnostic Computed tomography (CT) (71.4%; 35/49), and to a lesser extent, Positron emission tomography (PET/CT) imaging (44.9%; 22/49). For bone SBRT planning, a CT slice thickness > 1 but below 2 mm was recommended by 54.5% of participants (24/44), and 34.1% of ROs (15/44) recommended a slice thickness of ≤ 1 mm.

For the application of bone SBRT, the majority of ROs use conventional linear accelerators (46.3%; 19/41) most often, followed by conventional linear accelerators with X‑ray verification for SRT (37.2%; 16/43) and dedicated linear accelerators with external X‑ray verification for SRT (28.2%; 11/39). Only a minority of treating ROs use specialized systems such as MR-linear accelerators (8.6%; 3/35) and robotic radiosurgery (24.3%; 9/37) at least rarely.

For treatment verification, CT (cone-beam or fan-beam) imaging before treatment was reported as the commonly used modality (84.8%; 39/46), and additional CT after couch repositioning is also preferred by 42.5% of participating ROs (17/40).

**Fig. 4:**
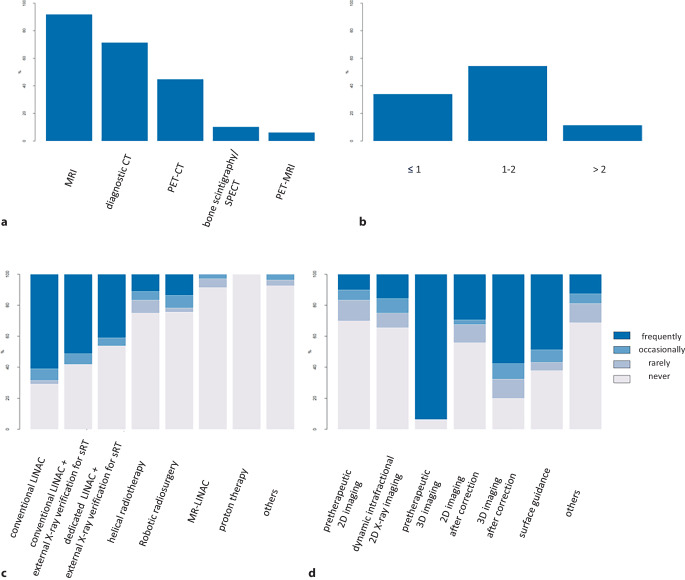
Patterns for treatment planning and application of bone stereotactic body radiotherapy (SBRT). **a** Imaging modalities required for treatment planning; **b** slice thickness for planning CT scans (in mm); **c** linear accelerator specifications used for bone SBRT: **d** treatment verification imaging used for bone SBRT (2D imaging including stereoscopic imaging)

Surface guidance/surveillance was used regularly by 48.6% of participating ROs (18/37; Fig. 4).

### Dose concepts and contouring for bone SBRT

Contouring and dose prescription concepts were separately recorded for spinal and non-spinal BoM. Most participating ROs stated the frequent use of compartment-based anatomic target volume concepts based on existing guidelines for spinal SBRT (59.5%; 25/42), and the frequent use of simultaneous integrated boost/dose-painting techniques was reported by 57.4% (27/47). Isotropic expansions of the gross tumor volume without a clinical target volume (25.5%; 12/47) or with a clinical target volume (15.6%; 7/45) or whole vertebral body treatment concepts (18.1%; 8/44) were less frequently used.

For non-spinal metastases, simultaneous integrated boost/dose-painting concepts were regularly prescribed by 44.7% of ROs (21/47), and isotropic GTV–PTV expansion was most commonly applied (51.2%; 23/45). In this context, the majority of ROs recommended a safety margin from the GTV to the PTV of 3–5 mm (63.1%; 12/19).

**Fig. 5:**
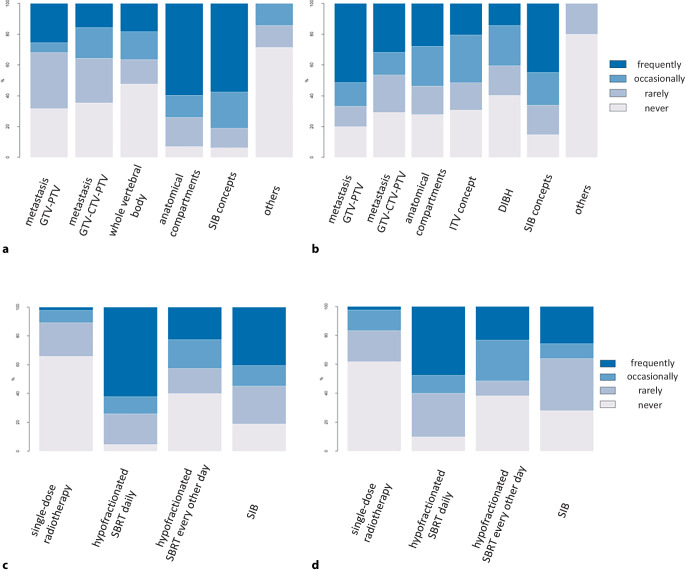
Dose concepts and contouring patterns for bone SBRT. Target volume concepts for SBRT of **a** spinal metastases and **b** non-spinal metastases; **c **dose concepts for SBRT of **c** spinal metastases and **d** non-spinal metastases

Dose and fractionation concepts varied considerably for both spinal and non-spinal SBRT. The use of single-fraction bone SBRT was rarely reported by participating ROs, with only 16.7% of ROs (7/42) using single-fraction treatments at least occasionally for non-spinal metastases and 10.6% of ROs (5/47) using single-fraction treatments at least occasionally for spinal metastases; 61.9% (26/42) and 66.0% (31/47) of participants ruled out the use of single-fraction bone SBRT for non-spinal and spinal metastases, respectively. Hypofractionated bone SBRT applied daily was the most frequently used method for both non-spinal (47.5%; 19/40) and spinal metastases (61.9%; 26/42; Fig. 5). Of participating ROs, 25.6% (10/39) and 40.5% (17/42) reported employing dose-painting concepts with integrated boost doses frequently for bone SBRT of non-spinal and spinal metastases, respectively.

The question about specific dose concepts for both spinal and non-spinal BoM was answered by too few participants for clear conclusions to be drawn.

## Discussion

In recent years, bone SBRT has been increasingly used in the context of OMD. SBRT might provide long-term freedom from progression or even a cure in highly selected patients in the case of total metastatic ablation, and some trials have shown a benefit in terms of progression-free survival and overall survival especially for limited BoM, while other trials have failed to replicate these findings for certain tumor types such as breast cancer [11–16]. Despite the increasing number of studies and published practice guidelines, little is known about the use of bone SBRT and the actual patterns of care for patients with bone oligometastases in German-speaking countries. A recent online survey in Germany on conventional and stereotactic RT for spinal metastases showed that SBRT was not comprehensively practiced, and the choice of dose regimen and target volume definition varied considerably between centers [24].

To our knowledge, our survey is the first patterns-of-care analysis for bone SBRT of spinal and non-spinal BoM in German-speaking countries. It demonstrates that bone SBRT is mostly performed for bone oligometastases. For symptom-directed palliative radiotherapy of BoM, the majority of responding physicians still mostly recommend moderately hypofractionated treatment concepts. Nevertheless, more than half of participating ROs stated that they use bone SBRT also for palliative purposes at least occasionally. Our survey also revealed a certain reluctance for the concomitant use of targeted agents, chemotherapy, or immunotherapy with bone SBRT.

In recent years, bone SBRT has become established as an ablative treatment for cases in which permanent control of local metastases was intended, and bone SBRT has been shown to prevent disease progression and improve cure rates in selected patients with bone OMD [7, 8, 11, 12, 20]. Therefore, it is not surprising that our patterns-of-care survey demonstrated a strong use of bone SBRT especially for OMD. On the other hand, bone SBRT for pain control and metastatic stabilization in the palliative setting is currently only employed by a portion of ROs and only for highly selected patients in German-speaking countries. This is in line with the current guideline from the Advisory Committee for Radiation Oncology Practice of the European Society for Radiotherapy and Oncology (ESTRO-ACROP) stating that palliatively dosed and fractionated radiotherapy remains the standard of care for treatment of symptomatic uncomplicated BoM, because of its significant lasting pain control with minimal toxicity [4, 26]. Nevertheless, in light of recent evidence, the ESTRO-ACROP guideline recommended consideration of highly conformal bone SBRT for selected patients [4, 27].

To date, at least six randomized trials have investigated the benefit of bone SBRT compared to conventional RT for spinal and non-spinal BoM, albeit with heterogeneous dose concepts and somewhat conflicting outcomes: with regard to the overall pain response rates at 3 months, the trials published by Ryu et al. and Sprave et al. in spinal BoM [28, 29], the trial by Nguyen et al. in non-spinal BoM [30], and the trial by Pielkenrood in a mixed group of BoM [31] failed to demonstrate a benefit of bone SBRT. Nevertheless, Sprave et al. could demonstrate a faster decrease in pain and significantly lower pain levels at 6 months following single-fraction SBRT for spinal metastases [29]. A similar benefit regarding a better pain response after 6 months was shown in the randomized DOSIS trial with dose-intensified five-fraction SBRT compared to conventional five-fraction RT [32]. Another randomized controlled phase II/III trial showed higher rates of complete pain response after two-fraction SBRT with 24 Gy compared to palliative RT with 20 Gy in five fractions for painful spinal BoM [33].

However, the available evidence does not yet fully support broad usage of bone SBRT for painful BoM in clinical routine, and further data are needed [4]. In light of this, the recently published practice guideline of the American Society for Radiation Oncology (ASTRO) also conditionally recommends bone SBRT over conventional palliative RT for symptomatic BoM in selected patients with good performance status and without an indication for surgery or neurologic symptoms [34].

In a recently published study on prophylactic RT of asymptomatic high-risk BoM, three-fraction bone SBRT was most commonly prescribed, and it can be concluded that this treatment reduced SREs and hospitalization rates; additionally, this trial even suggested an OS benefit for prophylactic palliative bone RT [35, 36]. However, due to the small sample size in this trial and the heterogeneity of dose fractionation concepts mixing SBRT and conventional fractionation, further phase III trials are needed to corroborate the demonstrated benefit and the low occurrence rates of radiotherapy-related SREs [37].

Mirroring the heterogeneous treatment concepts in this trial, our patterns-of-care analysis also demonstrated a strong heterogeneity in RT concepts for BoM in routine care in German-speaking countries. In our analysis, the use of single-fraction bone SBRT was less frequently reported by participating ROs, with “at least occasionally” stated by only 16.7% for non-spinal metastases and 10.6% for spinal metastases; two- to five-fraction bone SBRT with daily application was the most frequently used method for both spinal and non-spinal BoM, and dose-painting approaches with integrated boost doses seem very common, especially for SBRT of spinal metastases. The current ASTRO clinical practice guideline for palliation recommends single-fraction SBRT with 12 to 16 Gy for symptomatic non-spinal metastases and two fractions of 12 Gy each for spinal metastases but suggests that other concepts with comparable biologically equivalent doses (BED) may also be applicable for individual patients, depending on the primary tumor and normal tissue tolerance as well as the physician’s experience [34]. However, single-fraction bone SBRT with 24 Gy was not recommended for palliation due to the relatively high fracture rate of almost 30% at 6 months. Nevertheless, ablative single-fraction bone SBRT may be considered in selected cases of OMD where ablation is intended. This is also mirrored in the ESTRO-ACROP guideline stating that the goal of local control should be balanced with the higher risk of vertebral compression fractures; the guideline recommends single doses exceeding 18 Gy for ablative bone treatments [20].

In a recent meta-analysis of SBRT for spinal metastases, 69 studies with more than 5700 patients were analyzed. The meta-analysis demonstrated high efficacy and an impressive overall pain response rate of 83%, with local control rates exceeding 94% at 1 year, albeit with considerable heterogeneity among the included trials [38]. Bone SBRT was demonstrated to be safe, with vertebral fracture rates of 9% and radiation-induced myelopathy rates of 0%. In this meta-analysis, 79.9% of all studies specified the use of image-guided radiotherapy, with a strong focus on CBCT (72.7%). Encouragingly, the data from our patterns-of-care analysis regarding image guidance correspond well with these data: pretreatment CBCT was reported as the most commonly used imaging modality. However, in German-speaking countries, multifraction hypofractionated bone SBRT was the most frequently used approach for non-spinal and spinal metastases.

Regarding its relevance for systemic tumor control, our survey demonstrated that the concomitant application of systemic cancer agents with bone SBRT remains a point of concern for many ROs. Although data from the international TOaSTT database found no significant difference in severe toxicity for continuous targeted therapies combined with SBRT [39], only about one third of our participating ROs felt safe combining bone SBRT with both concomitant and sequential systemic treatments. In our patterns-of-care analysis, more than one third of treating ROs declined the concomitant use of targeted agents, chemotherapy, or immunotherapy with bone SBRT. Interestingly, many ROs reported also stopping hormone-modifying agents such as androgen deprivation therapy during bone SBRT. In light of these uncertainties and concerns, it is worth taking a look at a recently published review by the EORTC–ESTRO OligoCare consortium analyzing the risk of toxicities from SBRT combined with targeted therapy or immunotherapy and reporting overall grade 3 toxicity in 21% and grades 4 and 5 in 1% of patients each [40]. This comprehensive review formed the basis of a Delphi consensus by the EORTC–ESTRO OligoCare consortium on the safe combination of SBRT with various systemic therapies, although the experts reported a lack of high-level evidence here as well [40].

It has to be noted that our analysis has some limitations. Our survey was mostly answered by senior ROs and ROs in high-volume institutions. While this may provide a reliable database for the clinical workflow in large academic centers, our survey may not provide the patterns of care in smaller, potentially less experienced practices that may not participate in the DEGRO-WG RS and SRT but may still offer bone SBRT to their patients. Also, due to the anonymity of the responders and the limited number of participants in our survey, no statistically robust subgroup analyses could be carried out. Regarding the combination of bone SBRT with systemic therapy, it was beyond the scope of this survey to analyze specific practice patterns such as the desired time interval between systemic therapy and SBRT or the duration of treatment interruption.

Taken together, our survey demonstrates that bone SBRT for spinal and non-spinal metastases in OMD is broadly available in clinical routine care in German-speaking countries. A large heterogeneity regarding dose and fractionation concepts exists, and the use of bone SBRT for palliative treatment of symptomatic or high-risk BoM remains inconsistent. Due to the heterogeneity in the implementation of bone SBRT with regard to dose and treatment volume concepts as well as treatment planning and application, further standardization of bone SBRT is required.

## Supplementary Information

All questions of the survey and frequency of corresponding answers are shown in supplementary material.

## Data Availability

The dataset generated during the current study is available from the corresponding author upon reasonable request
