# Supplementary material for: Stereotactic radiotherapy for spinal and non-spinal bone metastases: a patterns-of-care analysis in German-speaking countries as part of a project of the interdisciplinary Radiosurgery and Stereotactic Radiotherapy Working Group of the DEGRO/DGMP
Source: Strahlenther Onkol. 2025 Mar 18;202(1):74–83. doi: 10.1007/s00066-025-02387-y (PMC12819525; doi:10.1007/s00066-025-02387-y)
Supplement: Supplementary file 1 — All questions of the survey and frequency of corresponding answers are shown in supplementary material. [file 66_2025_2387_MOESM1_ESM.docx]

**Supplementary Data**

**Supplementary Fig. 1** Participating centers and position of the participants within their radiotherapy center


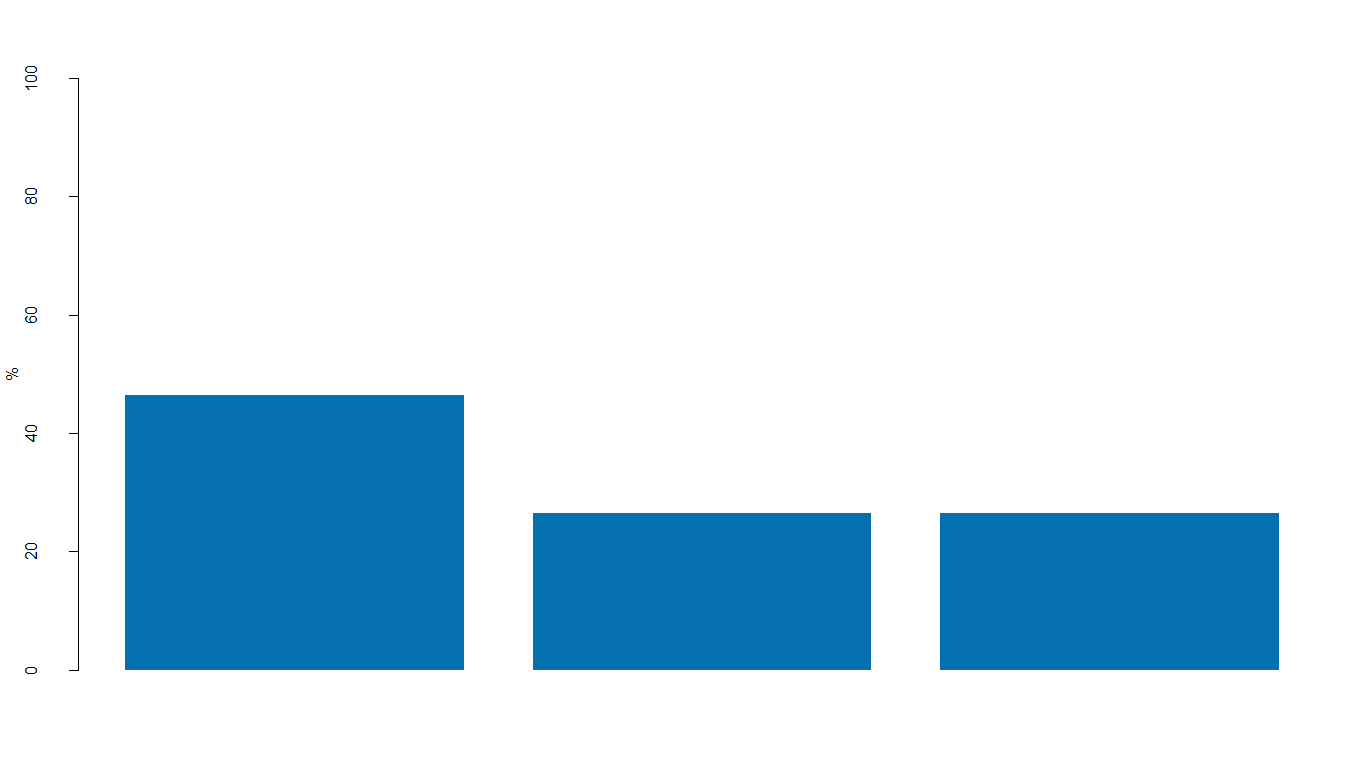

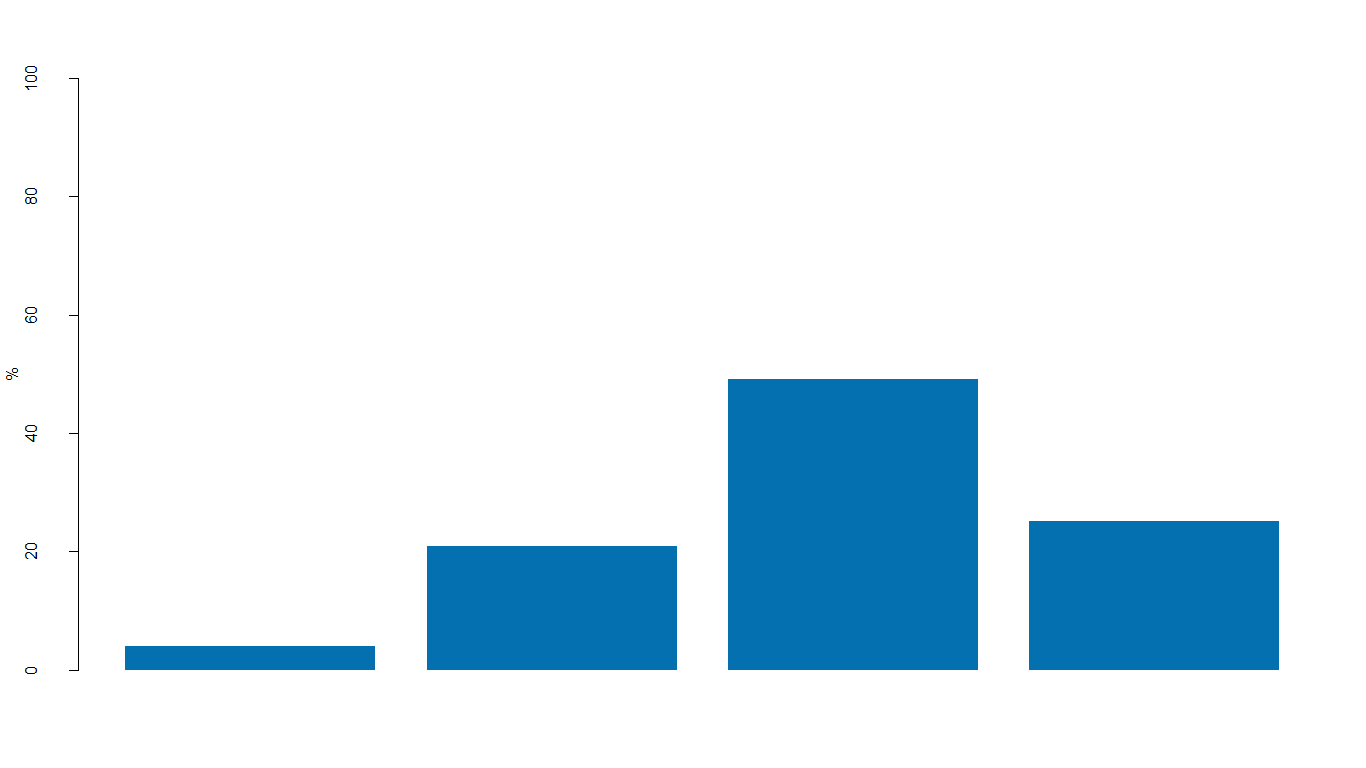


university

hospital

non-university

hospital

practice /

outpatient center

assistant physician “Assistenzarzt”

radiation oncologist “Facharzt”

Senior consultant physician “Oberarzt”

Departmental head “Chefarzt”

**Supplementary Fig. 2** General characteristics of the participating centers. (A) Number of patients treated for bone metastases per year. (B) Proportion of patients treated with stereotactic radiotherapy for BoM per year in %. (C) Treatment concepts used for palliative radiotherapy of BoM


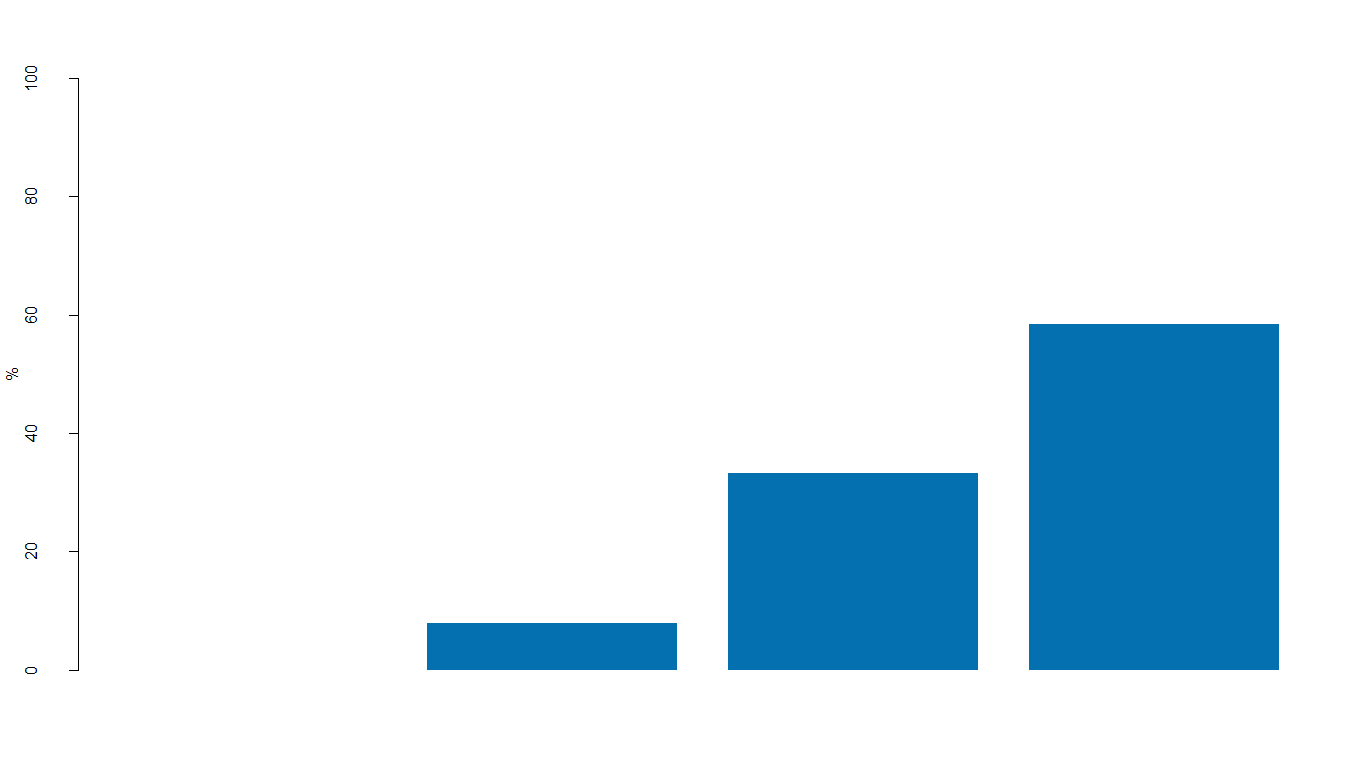

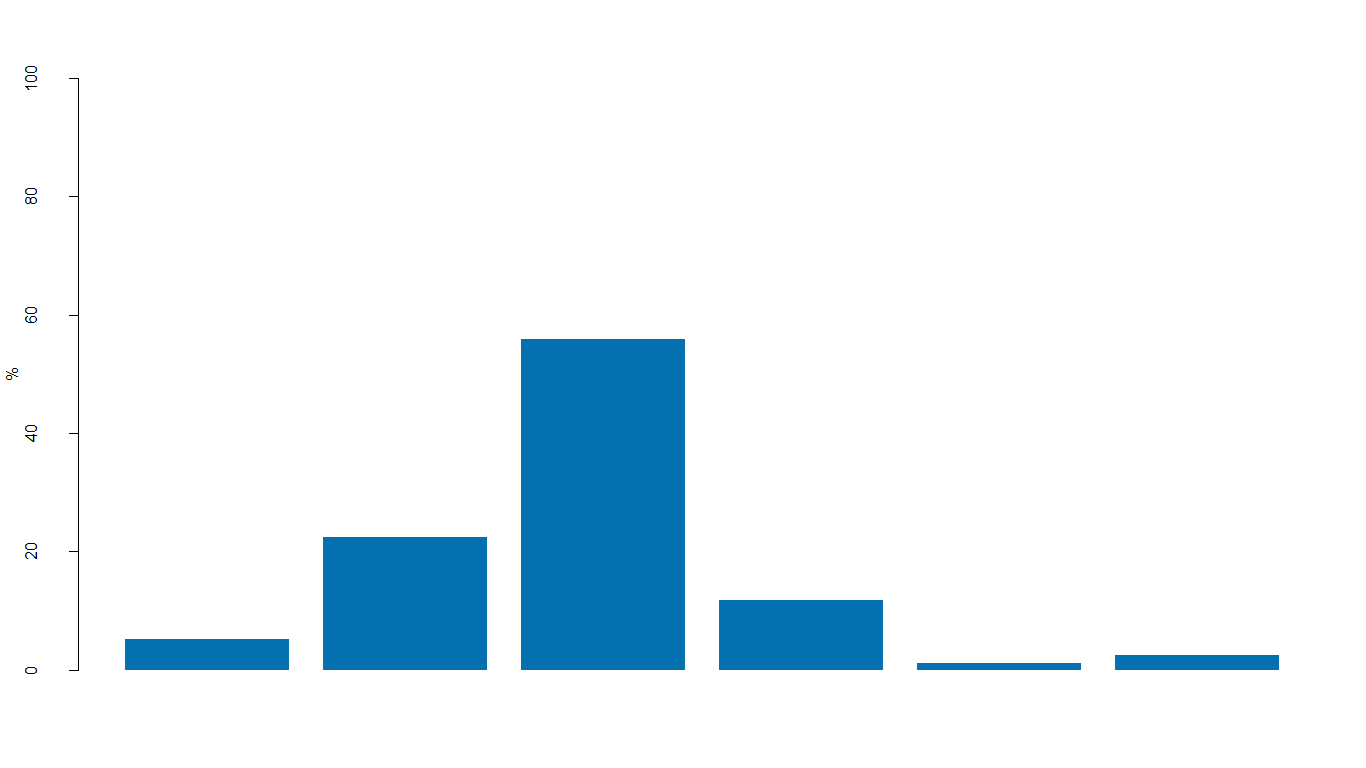

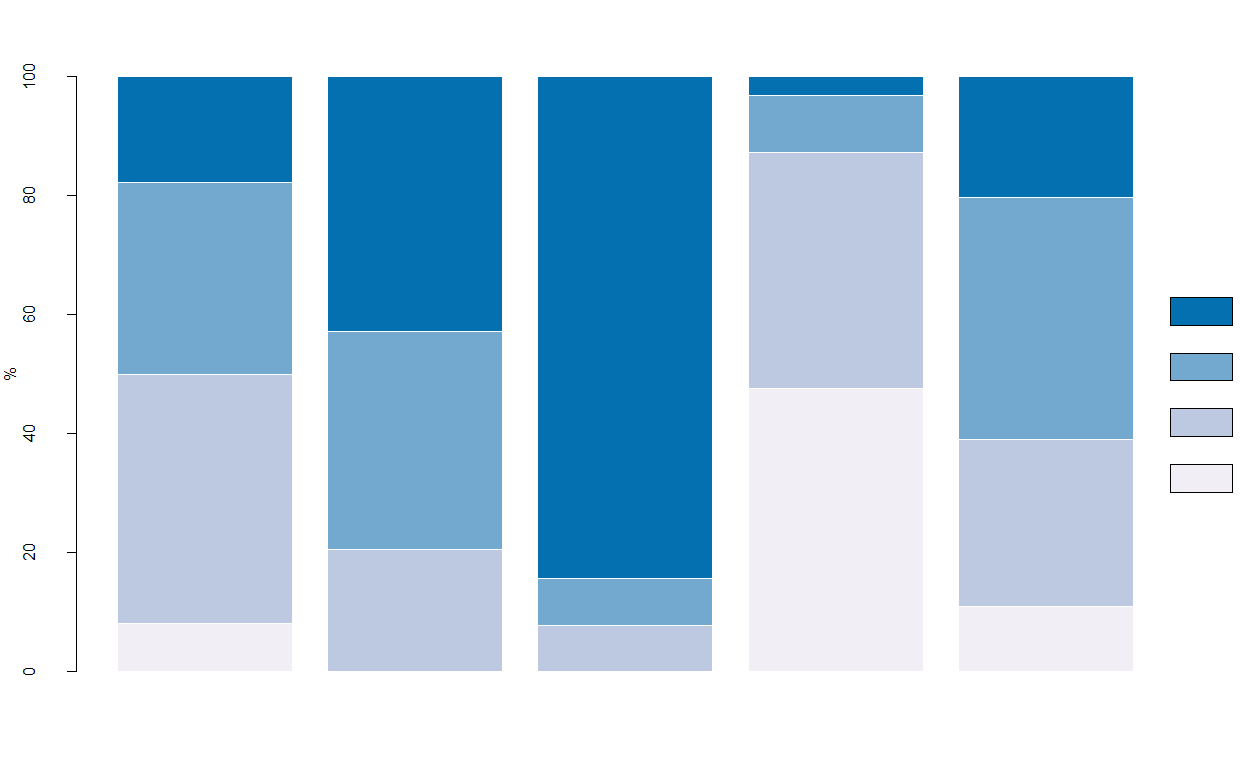


**A**

**B**

< 10

10-50

51-100

> 100

none

< 10 %

10-30 %

31-50 %

51-75 %

> 75 %

**C**

frequently

occasionally

rarely

never

single-dose

e.g. 1x 8 Gy

greatly hypofractionated e.g. 5x 4 Gy

moderately hypo-fractionated e.g. 10x 3 Gy

normofractionated e.g. 20x 2 Gy

stereotactically

ablative radiotherapy

**Supplementary Fig. 3** Comparison of treatment patterns between university and non-university treatment facilities. (A) Number of patients treated for bone metastases per year. (B) Proportion of patients treated with stereotactic radiotherapy for BoM per year in %. (C) Treatment concepts used for palliative radiotherapy of BoM


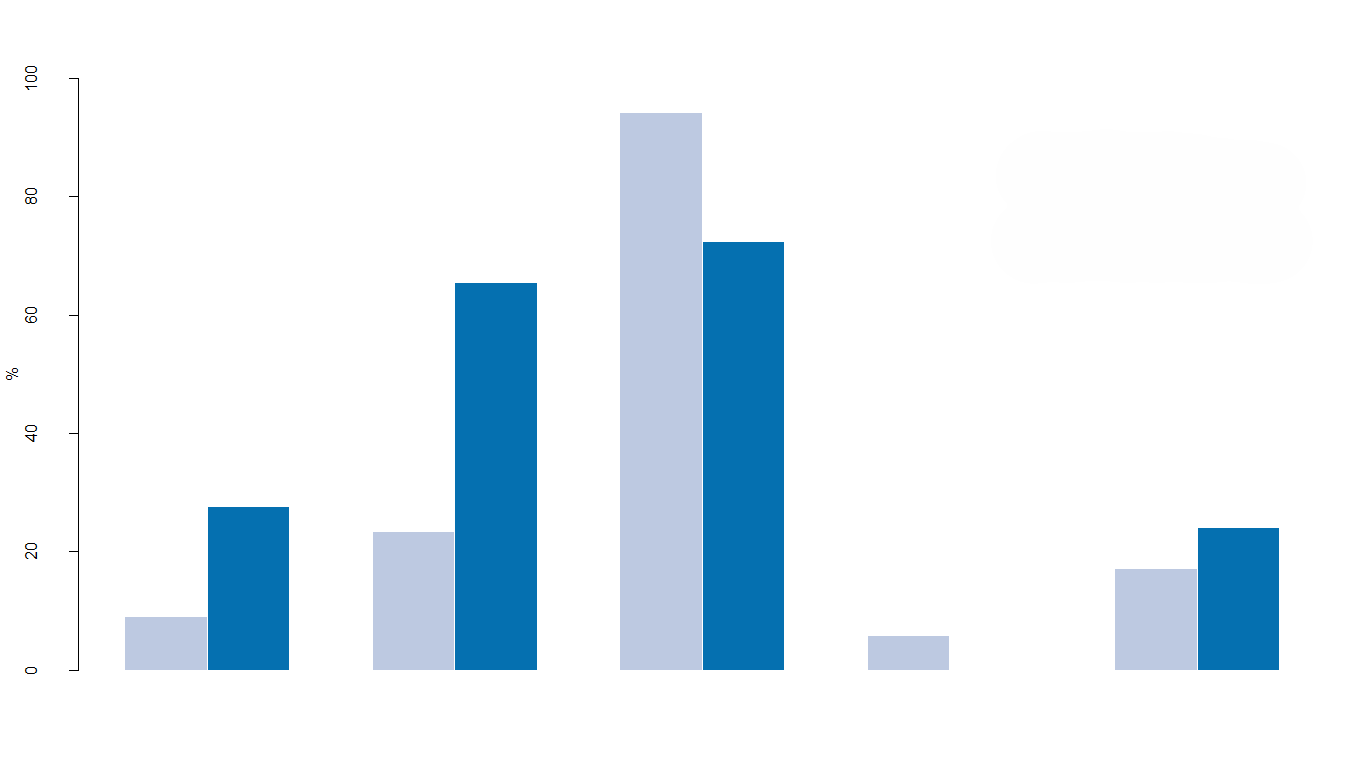

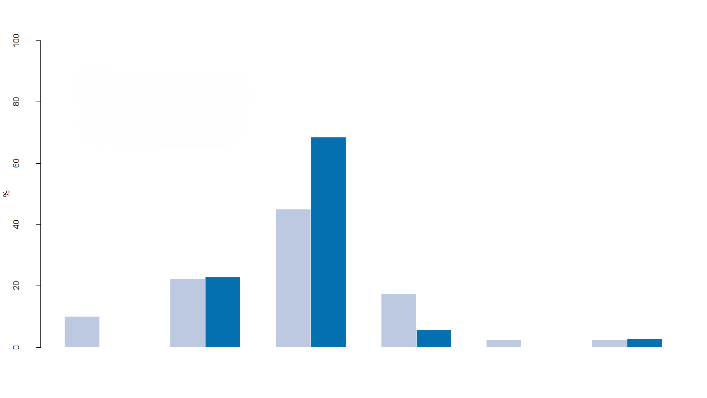

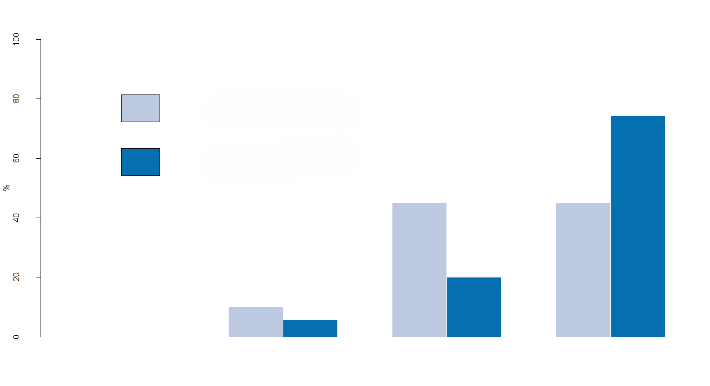


**A**

< 10

10-50

51-100

> 100

none

< 10 %

10-30 %

31-50 %

51-75 %

> 75 %

non-university facility

university hospital

**B**

*p < 0.04

* p = 0.03

* p = 0.001

**C**

single-dose

e.g. 1x 8 Gy

greatly hypofractionated e.g. 5x 4 Gy

moderately hypo-fractionated e.g. 10x 3 Gy

normofractionated e.g. 20x 2 Gy

stereotactically

ablative radiotherapy

**Supplementary Table 1** Summary of all questions and answers of the online-questionnaire.

stereotactic body radiotherapy (SBRT), bone metastases (BoM)

| **A General Data** | | **N** | | | **%** | | | | | | |
| --- | --- | --- | --- | --- | --- | --- | --- | --- | --- | --- | --- |
| **1 Type of radiotherapy facility** | |  | | |  | | | | | | |
| University hospital | | 35/75 | | | 46.7 % | | | | | | |
| Non-university hospital | | 20/75 | | | 26.7 % | | | | | | |
| Practice/ outpatient center | | 20/75 | | | 26.7 % | | | | | | |
| **2 Position in radiotherapy facility** | |  | | |  | | | | | | |
| Assistant physician (“Assistenzarzt”) | | 3/71 | | | 4.2 % | | | | | | |
| Board-certified radiation oncologist (“Facharzt”) | | 15/71 | | | 21.1 % | | | | | | |
| Senior consultant physician (“Oberarzt”) | | 35/71 | | | 49.3 % | | | | | | |
| Departmental head (“Chefarzt”) | | 18/71 | | | 25.4 % | | | | | | |
| **3 Number of treated patients with BoM per year** | |  | | |  | | | | | | |
| < 10 | | 0/75 | | | 0.0 % | | | | | | |
| 10-50 | | 6/75 | | | 8.0 % | | | | | | |
| 51-100 | | 25/75 | | | 33.3 % | | | | | | |
| > 100 | | 44/75 | | | 58.7 % | | | | | | |
| **4 Proportion of patients with BoM treated with SBRT** | |  | | |  | | | | | | |
| 0 % | | 4/75 | | | 5.3 % | | | | | | |
| < 10 % | | 17/75 | | | 22.7 % | | | | | | |
| 10-30 % | | 42/75 | | | 56.0 % | | | | | | |
| 31-50 % | | 9/75 | | | 12.0 % | | | | | | |
| 51-75 % | | 1/75 | | | 1.2 % | | | | | | |
| > 75 % | | 2/75 | | | 2.7 % | | | | | | |
| **B Medical Indications** | | **N** | | | **%** | | | | | | |
| **5 Regularly used concept for palliative radiotherapy of BoM** |  | | | |  | | | | | | |
| *palliative single-dose RT (e.g. 1x 8 Gy)* | |  | | |  | | | | | | |
| Never | | 5/62 | | | 8.1 % | | | | | | |
| Very rarely | | 19/62 | | | 30.7 % | | | | | | |
| Rarely | | 7/62 | | | 11.3 % | | | | | | |
| Occasionally | | 20/62 | | | 32.3 % | | | | | | |
| Frequently | | 9/62 | | | 14.5 % | | | | | | |
| Most frequently | | 2/62 | | | 3.2 % | | | | | | |
| *greatly hypofractionated with single dose ≥ 4 Gy (e.g. 5x 4 Gy)* | |  | | |  | | | | | | |
| Never | | 0/63 | | | 0.0 % | | | | | | |
| Very rarely | | 3/63 | | | 4.8 % | | | | | | |
| Rarely | | 10/63 | | | 15.9 % | | | | | | |
| Occasionally | | 23/63 | | | 36.5 % | | | | | | |
| Frequently | | 20/63 | | | 31.8 % | | | | | | |
| Most frequently | | 7/63 | | | 11.1 % | | | | | | |
| *moderately hypofractionated with single dose 2,5 - 3,5 Gy (e.g. 10-12x 3 Gy)* | | | | |  | | |  | | | |
| Never | | 0/64 | | | 0.0 % | | | | | | |
| Very rarely | | 1/64 | | | 1.6 % | | | | | | |
| Rarely | | 4/64 | | | 6.3 % | | | | | | |
| Occasionally | | 5/64 | | | 7.8 % | | | | | | |
| Frequently | | 15/64 | | | 23.4 % | | | | | | |
| Most frequently | | 39/64 | | | 60.9 % | | | | | | |
| *conventionally fractionated with single dose 2 Gy (e.g. 20x 2 Gy)* |  | | | | | |  | | | | |
| Never | | 30/63 | | | 47.6 % | | | | | | |
| Very rarely | | 19/63 | | | 30.2 % | | | | | | |
| Rarely | | 6/63 | | | 9.5 % | | | | | | |
| Occasionally | | 6/63 | | | 9.5 % | | | | | | |
| Frequently | | 2/63 | | | 3.2 % | | | | | | |
| Most frequently | | 0/63 | | | 0.0 % | | | | | | |
| *stereotactically ablative RT* | |  | | |  | | | | | | |
| Never | | 7/64 | | | 10.9 % | | | | | | |
| Very rarely | | 8/64 | | | 12.5 % | | | | | | |
| Rarely | | 10/64 | | | 15.6 % | | | | | | |
| Occasionally | | 26/64 | | | 40.6 % | | | | | | |
| Frequently | | 11/64 | | | 17.2 % | | | | | | |
| Most frequently | | 2/64 | | | 3.1 % | | | | | | |
| **6 Objective for treating BoM with SBRT** | |  | | |  | | | | | | |
| *local ablative therapy for oligometastasis/-progression* | |  | | |  | | | | | | |
| Never | | 2/61 | | | 3.3 % | | | | | | |
| Very rarely | | 2/61 | | | 3.3 % | | | | | | |
| Rarely | | 2/61 | | | 3.3 % | | | | | | |
| Occasionally | | 7/61 | | | 11.5 % | | | | | | |
| Frequently | | 13/61 | | | 21.3 % | | | | | | |
| Most frequently | | 35/61 | | | 57.4 % | | | | | | |
| *palliative for pain* | |  | | |  | | | | | | |
| Never | | 7/61 | | | 11.5 % | | | | | | |
| Very rarely | | 11/61 | | | 18.0 % | | | | | | |
| Rarely | | 12/61 | | | 19.7 % | | | | | | |
| Occasionally | | 19/61 | | | 31.1 % | | | | | | |
| Frequently | | 9/61 | | | 14.8 % | | | | | | |
| Most frequently | | 3/61 | | | 4.9 % | | | | | | |
| *palliative for (imminent) neurological symptoms* | |  | | |  | | | | | | |
| Never | | 17/61 | | | 27.9 % | | | | | | |
| Very rarely | | 11/61 | | | 18.0 % | | | | | | |
| Rarely | | 11/61 | | | 18.0 % | | | | | | |
| Occasionally | | 11/61 | | | 18.0 % | | | | | | |
| Frequently | | 9/61 | | | 14.8 % | | | | | | |
| Most frequently | | 2/61 | | | 3.3 % | | | | | | |
| *palliative for instability* | |  | | |  | | | | | | |
| Never | | 27/61 | | | 44.3 % | | | | | | |
| Very rarely | | 11/61 | | | 18.0 % | | | | | | |
| Rarely | | 11/61 | | | 18.0 % | | | | | | |
| Occasionally | | 7/61 | | | 11.5 % | | | | | | |
| Frequently | | 3/61 | | | 4.9 % | | | | | | |
| Most frequently | | 2/61 | | | 3.3 % | | | | | | |
| *prophylactic (early palliation)* | |  | | |  | | | | | | |
| Never | | 12/61 | | | 19.7 % | | | | | | |
| Very rarely | | 13/61 | | | 21.3 % | | | | | | |
| Rarely | | 11/61 | | | 18.0 % | | | | | | |
| Occasionally | | 17/61 | | | 27.3 % | | | | | | |
| Frequently | | 6/61 | | | 9.8 % | | | | | | |
| Most frequently | | 2/61 | | | 3.3 % | | | | | | |
| *Postoperative* | |  | | |  | | | | | | |
| Never | | 31/61 | | | 50.8 % | | | | | | |
| Very rarely | | 7/61 | | | 11.5 % | | | | | | |
| Rarely | | 10/61 | | | 16.4 % | | | | | | |
| Occasionally | | 10/61 | | | 16.4 % | | | | | | |
| Frequently | | 2/61 | | | 3.3 % | | | | | | |
| Most frequently | | 1/61 | | | 1.6 % | | | | | | |
| **7 Definition of bone oligometastasis?** | |  | | |  | | | | | | |
| 1 bone metastasis | | 1/63 | | | 1.6 % | | | | | | |
| At most 3 bone metastases | | 17/63 | | | 27.0 % | | | | | | |
| At most 5 bone metastases | | 26/63 | | | 41.3 % | | | | | | |
| Varying dependent on clinical/anatomical/histological factors | | 19/63 | | | 30.2 % | | | | | | |
| **8 Localizations amenable for bone SBRT** | | | | | | | | |  | |  |
| *cervical spine* | |  | | |  | | | | | | |
| Never | | 4/61 | | | 6.6 % | | | | | | |
| Very rarely | | 8/61 | | | 13.1 % | | | | | | |
| Rarely | | 12/61 | | | 19.7 % | | | | | | |
| Occasionally | | 21/61 | | | 34.4 % | | | | | | |
| Frequently | | 14/61 | | | 22.9 % | | | | | | |
| Most frequently | | 2/61 | | | 3.3 % | | | | | | |
| *thoracic/lumbar spine* | |  | | |  | | | | | | |
| Never | | 2/62 | | | 3.2 % | | | | | | |
| Very rarely | | 2/62 | | | 3.2 % | | | | | | |
| Rarely | | 2/62 | | | 3.2 % | | | | | | |
| Occasionally | | 27/62 | | | 43.5 % | | | | | | |
| Frequently | | 18/62 | | | 29.0 % | | | | | | |
| Most frequently | | 11/62 | | | 17.7 % | | | | | | |
| *thorax (ribs/scapula/sternum)* | |  | | |  | | | | | | |
| Never | | 2/60 | | | 3.3 % | | | | | | |
| Very rarely | | 2/60 | | | 3.3 % | | | | | | |
| Rarely | | 5/60 | | | 8.3 % | | | | | | |
| Occasionally | | 27/60 | | | 45.0 % | | | | | | |
| Frequently | | 23/60 | | | 38.3 % | | | | | | |
| Most frequently | | 1/60 | | | 1.7 % | | | | | | |
| *Pelvis* | |  | | |  | | | | | | |
| Never | | 1/62 | | | 1.6 % | | | | | | |
| Very rarely | | 2/62 | | | 3.2 % | | | | | | |
| Rarely | | 4/62 | | | 6.5 % | | | | | | |
| Occasionally | | 23/62 | | | 37.1 % | | | | | | |
| Frequently | | 28/62 | | | 45.2 % | | | | | | |
| Most frequently | | 4/62 | | | 6.4 % | | | | | | |
| *long tubular bones* | |  | | |  | | | | | | |
| Never | | 9/62 | | | 14.5 % | | | | | | |
| Very rarely | | 11/62 | | | 17.7 % | | | | | | |
| Rarely | | 12/62 | | | 19.4 % | | | | | | |
| Occasionally | | 20/62 | | | 32.3 % | | | | | | |
| Frequently | | 10/62 | | | 16.1 % | | | | | | |
| Most frequently | | 0/62 | | | 0.0 % | | | | | | |
| *others (e.g. calvaria)* | |  | | |  | | | | | | |
| Never | | 8/21 | | | 38.1 % | | | | | | |
| Very rarely | | 6/21 | | | 28.6 % | | | | | | |
| Rarely | | 2/21 | | | 9.5 % | | | | | | |
| Occasionally | | 3/21 | | | 14.3 % | | | | | | |
| Frequently | | 2/21 | | | 9.5 % | | | | | | |
| Most frequently | | 0/21 | | | 0.0 % | | | | | | |
| **9 Histology-based decisions for/against bone SBRT** | | |  | |  | | | | | | |
| Yes | | 24/61 | | | 39.3 % | | | | | | |
| No | | 37/61 | | | 60.7 % | | | | | | |
| **10 Histologies for which bone SBRT is not recommended** | | | | |  | | | | |  | |
| Neuroendocrine tumors (SCLC) | | 16/51 | | | 31.4 % | | | | | | |
| Multiple Myeloma | | 11/51 | | | 21.6 % | | | | | | |
| Hematological diseases | | 14/51 | | | 27.5 % | | | | | | |
| Others | | 10/51 | | | 19.6 % | | | | | | |
| **11 Absolute contraindications for SBRT of BoM** | |  | | |  | | | | | | |
| Instability of the bone with necessity for operative stabilization | | 50/58 | | | 86.2 % | | | | | | |
| Relevant infiltration of the spinal canal | | 26/58 | | | 44.8 % | | | | | | |
| Limited life expectancy of < 3 months | | 23/58 | | | 39.7 % | | | | | | |
| New or aggravating neurological symptoms | | 22/58 | | | 37.9 % | | | | | | |
| Direct contact with structures at risk | | 16/58 | | | 27.6 % | | | | | | |
| Pre-irradiation in loco | | 7/58 | | | 12.1 % | | | | | | |
| Lacking eligibility of the patient for MRI | | 6/58 | | | 10.3 % | | | | | | |
| Others | | 2/58 | | | 3.4 % | | | | | | |
| **12 Continuation of systemic therapy during SBRT of BoM** | | | | | |  | | | |  | |
| yes – concomitantly | | 19/62 | | | 30.6 % | | | | | | |
| yes – sequentially | | 20/62 | | | 32.3 % | | | | | | |
| yes - concomitantly and sequentially | | 20/62 | | | 32.3 % | | | | | | |
| no (e.g. exclusively ablative RT of all manifestations) | | 3/62 | | | 4.9 % | | | | | | |
| **13 Systemic therapies with no concomitant use of bone SBRT** | | |  | | | |  | | | | |
| targeted therapies | | 27/42 | | | 64.3 % | | | | | | |
| Chemotherapy | | 22/42 | | | 52.4 % | | | | | | |
| Immunotherapy | | 19/42 | | | 45.2 % | | | | | | |
| hormone therapy (ADT/SIRM) | | 13/42 | | | 30.9 % | | | | | | |
| other substances | | 6/42 | | | 14.3 % | | | | | | |
| *Specific substances* | |  | | |  | | | | | | |
| BRAF/ MEK inhibitors | | 6/12 | | | 50.0 % | | | | | | |
| VEGF inhibitors | | 5/12 | | | 41.7 % | | | | | | |
| PARP inhibitors | | 3/12 | | | 25.0 % | | | | | | |
| TKI (general) | | 2/12 | | | 16.7 % | | | | | | |
| MTX | | 1/12 | | | 8.3 % | | | | | | |
| CDK4/6 inhibitors | | 1/12 | | | 8.3 % | | | | | | |
| **14 Fraction of patients receiving bone-modifying drugs** | |  | | |  | | | | | | |
| < 25 % | | 5/62 | | | 8.1 % | | | | | | |
| 25-50 % | | 22/62 | | | 35.5 % | | | | | | |
| 51-90 % | | 23/62 | | | 37.1 % | | | | | | |
| > 90 % | | 12/62 | | | 19.4 % | | | | | | |
| **15 Postoperative SBRT after resection of BoM** | |  | | |  | | | | | | |
| No | | 45/63 | | | 71.4 % | | | | | | |
| Yes - after dorsal stabilization of the spine with partial resection of the metastasis | | 7/63 | | | 11.1 % | | | | | | |
| Yes – after incomplete resection of bone metastases | | 8/63 | | | 12.7 % | | | | | | |
| Yes - after complete resection of a bone metastasis | | 0/63 | | | 0.0 % | | | | | | |
| Alternative | | 3/63 | | | 4.8 % | | | | | | |
| **C Planning of Stereotactic Radiotherapy** | | **n** | | | **%** | | | | | | |
| **16 Used immobilization for SBRT of bone metastases** | |  | | |  | | | | | | |
| Conventional measures for positioning (e.g. conventional masks, knee pillows) | | 32/49 | | | 65.3 % | | | | | | |
| Special measures for positioning or fixation, e.g. vacuum mats, masks for SRT | | 39/49 | | | 79.6 % | | | | | | |
| Motion management for breathing-dependent target volumes (e.g. respiratory gating, abdominal pressing) | | 27/49 | | | 55.1 % | | | | | | |
| **17 Used planning systems** | |  | | |  | | | | | | |
| Eclipse | | 31/49 | | | 63.3 % | | | | | | |
| Monaco | | 8/49 | | | 16.3 % | | | | | | |
| Precision | | 8/49 | | | 16.3 % | | | | | | |
| Raystation | | 7/49 | | | 14.3 % | | | | | | |
| Brainlab Elements | | 5/49 | | | 10.2 % | | | | | | |
| Pinnacle | | 5/49 | | | 10.2 % | | | | | | |
| PET-CT | | 1/49 | | | 2.0 % | | | | | | |
| **18 Additional imaging routinely required for planning** | |  | | |  | | | | | | |
| MRI | | 45/49 | | | 91.9 % | | | | | | |
| diagnostic CT | | 35/49 | | | 71.4 % | | | | | | |
| PET-CT | | 22/49 | | | 44.9 % | | | | | | |
| bone scintigraphy/SPECT | | 5/49 | | | 10.2 % | | | | | | |
| PET-MRI | | 3/49 | | | 6.1 % | | | | | | |
| *Planning CT scan – layer thickness (in mm)* | |  | | |  | | | | | | |
| ≤ 1 | | 15/44 | | | 34.1 % | | | | | | |
| 1-2 | | 24/44 | | | 54.5 % | | | | | | |
| > 2 | | 5/44 | | | 11.4 % | | | | | | |
| **19 Predominant target volume concept for spine SBRT** | |  | | |  | | | | | | |
| *Solely metastasis with exclusive expansion of GTV to PTV as GTV-PTV concept* | |  | | |  | | | | | | |
| Never | | 15/47 | | | 31.9 % | | | | | | |
| Very rarely | | 8/47 | | | 17.0 % | | | | | | |
| Rarely | | 9/47 | | | 19.1 % | | | | | | |
| Occasionally | | 3/47 | | | 6.4 % | | | | | | |
| Frequently | | 5/47 | | | 10.6 % | | | | | | |
| Most frequently | | 7/47 | | | 14.9 % | | | | | | |
| *Safety margin (mm)* | |  | | |  | | | | | | |
| ≤ 2 | | 7/19 | | | 36.8 % | | | | | | |
| 2-3 | | 5/19 | | | 26.3 % | | | | | | |
| 3-5 | | 7/19 | | | 36.8 % | | | | | | |
| *Solely metastasis with GTV-CTV-PTV concept with isotropic margin* | |  | | |  | | | | | | |
| Never | | 16/45 | | | 35.5 % | | | | | | |
| Very rarely | | 10/45 | | | 22.2 % | | | | | | |
| Rarely | | 3/45 | | | 6.7 % | | | | | | |
| Occasionally | | 9/45 | | | 20.0 % | | | | | | |
| Frequently | | 3/45 | | | 6.7 % | | | | | | |
| Most frequently | | 4/45 | | | 8.9 % | | | | | | |
| *Safety margin (mm)* | |  | | |  | | | | | | |
| ≤ 2 | | 5/13 | | | 38.5 % | | | | | | |
| 2-3 | | 6/13 | | | 46.1 % | | | | | | |
| 3-5 | | 0/13 | | | 0.0  % | | | | | | |
| 10 | | 2/13 | | | 15.4 % | | | | | | |
| *Whole vertebral body* | |  | | |  | | | | | | |
| Never | | 21/44 | | | 47.7 % | | | | | | |
| Very rarely | | 4/44 | | | 9.1 % | | | | | | |
| Rarely | | 3/44 | | | 6.8 % | | | | | | |
| Occasionally | | 8/44 | | | 18.2 % | | | | | | |
| Frequently | | 5/44 | | | 11.3 % | | | | | | |
| Most frequently | | 3/44 | | | 6.8 % | | | | | | |
| *Safety margin (mm)* | |  | | |  | | | | | | |
| ≤ 2 | | 6/9 | | | 66.7 % | | | | | | |
| 2-3 | | 2/9 | | | 22.2 % | | | | | | |
| 3-5 | | 1/9 | | | 11.1 % | | | | | | |
| *Anatomical concepts (“compartments“) according to existing consensus guidelines/recommendations* | |  | | |  | | | | | | |
| Never | | 3/42 | | | 7.1 % | | | | | | |
| Very rarely | | 3/42 | | | 7.1 % | | | | | | |
| Rarely | | 5/42 | | | 11.9 % | | | | | | |
| Occasionally | | 6/42 | | | 14.3 % | | | | | | |
| Frequently | | 9/42 | | | 21.4 % | | | | | | |
| Most frequently | | 16/42 | | | 38.1 % | | | | | | |
| *Concept* | |  | | |  | | | | | | |
| DOSIS study | | 5/8 | | | 62.5 % | | | | | | |
| PREST | | 1/8 | | | 12.5 % | | | | | | |
| RTOG | | 1/8 | | | 12.5 % | | | | | | |
| SPINAL | | 1/8 | | | 12.5 % | | | | | | |
| *Concepts with SIB/protection* | |  | | |  | | | | | | |
| Never | | 3/47 | | | 6.4 % | | | | | | |
| Very rarely | | 3/47 | | | 6.4 % | | | | | | |
| Rarely | | 3/47 | | | 6.4 % | | | | | | |
| Occasionally | | 11/47 | | | 23.4 % | | | | | | |
| Frequently | | 8/47 | | | 17.0 % | | | | | | |
| Most frequently | | 19/47 | | | 40.4 % | | | | | | |
| *Others* | |  | | |  | | | | | | |
| Never | | 5/7 | | | 71.4 % | | | | | | |
| Very rarely | | 0/7 | | | 0.0 % | | | | | | |
| Rarely | | 1/7 | | | 14.3 % | | | | | | |
| Occasionally | | 1/7 | | | 14.3 % | | | | | | |
| Frequently | | 0/7 | | | 0.0 % | | | | | | |
| Most frequently | | 0/7 | | | 0.0 % | | | | | | |
| **20 Predominant target volume concept for non-spine SBRT** | |  | | |  | | | | | | |
| *Solely metastasis with exclusive expansion of GTV to PTV as GTV-PTV concept* | |  | | |  | | | | | | |
| Never | | 9/45 | | | 20.0 % | | | | | | |
| Very rarely | | 4/45 | | | 8.9 % | | | | | | |
| Rarely | | 2/45 | | | 4.4 % | | | | | | |
| Occasionally | | 7/45 | | | 15.6 % | | | | | | |
| Frequently | | 11/45 | | | 24.5 % | | | | | | |
| Most frequently | | 12/45 | | | 26.7 % | | | | | | |
| *Safety margin (mm)* | |  | | |  | | | | | | |
| ≤ 2 | | 2/19 | | | 10.5 % | | | | | | |
| 2-3 | | 4/19 | | | 21.1 % | | | | | | |
| 3-5 | | 12/19 | | | 63.1 % | | | | | | |
| 10 | | 1/19 | | | 5.3 % | | | | | | |
| *Solely metastasis with GTV-CTV-PTV concept with isotropic margin* | |  | | |  | | | | | | |
| Never | | 12/41 | | | 29.3 % | | | | | | |
| Very rarely | | 8/41 | | | 19.5 % | | | | | | |
| Rarely | | 2/41 | | | 4.9 % | | | | | | |
| Occasionally | | 6/41 | | | 14.6 % | | | | | | |
| Frequently | | 7/41 | | | 17.1 % | | | | | | |
| Most frequently | | 6/41 | | | 14.6 % | | | | | | |
| *Safety margin (mm)* | |  | | |  | | | | | | |
| ≤ 2 | | 5/12 | | | 41.7 % | | | | | | |
| 2-3 | | 1/12 | | | 8.3 % | | | | | | |
| 3-5 | | 4/12 | | | 33.3 % | | | | | | |
| 10 | | 2/12 | | | 16.7 % | | | | | | |
| *Anatomical concepts (“compartments“) according to existing consensus guidelines/recommendations* | |  | | |  | | | | | | |
| Never | | 12/43 | | | 27.9 % | | | | | | |
| Very rarely | | 4/43 | | | 9.3 % | | | | | | |
| Rarely | | 4/43 | | | 9.3 % | | | | | | |
| Occasionally | | 11/43 | | | 25.6 % | | | | | | |
| Frequently | | 6/43 | | | 14.0 % | | | | | | |
| Most frequently | | 6/43 | | | 14.0 % | | | | | | |
| *ITV concept* | |  | | |  | | | | | | |
| Never | | 12/39 | | | 30.8 % | | | | | | |
| Very rarely | | 5/39 | | | 12.8 % | | | | | | |
| Rarely | | 2/39 | | | 5.1 % | | | | | | |
| Occasionally | | 12/39 | | | 30.8 % | | | | | | |
| Frequently | | 8/39 | | | 20.5 % | | | | | | |
| Most frequently | | 0/39 | | | 0.0 % | | | | | | |
| *Free-text* | |  | | |  | | | | | | |
| Ribs | | 4/6 | | | 66.7 % | | | | | | |
| Abdominal pressing | | 1/6 | | | 16.7 % | | | | | | |
| Sternum | | 1/6 | | | 16.7 % | | | | | | |
| *DIBH, e.g. for thoracic metastases* | |  | | |  | | | | | | |
| Never | | 17/42 | | | 40.5 % | | | | | | |
| Very rarely | | 3/42 | | | 7.1 % | | | | | | |
| Rarely | | 5/42 | | | 11.9 % | | | | | | |
| Occasionally | | 11/42 | | | 26.2 % | | | | | | |
| Frequently | | 5/42 | | | 11.9 % | | | | | | |
| Most frequently | | 1/42 | | | 2.4 % | | | | | | |
| *Safety margin CTV/ PTV* | |  | | |  | | | | | | |
| ≤ 2 | | 2/13 | | | 15.4 % | | | | | | |
| 2-3 | | 7/13 | | | 53.8 % | | | | | | |
| 3-5 | | 4/13 | | | 30.1 % | | | | | | |
| *Concepts with SIB/protection* | |  | | |  | | | | | | |
| Never | | 7/47 | | | 14.9 % | | | | | | |
| Very rarely | | 3/47 | | | 6.4 % | | | | | | |
| Rarely | | 6/47 | | | 12.8 % | | | | | | |
| Occasionally | | 10/47 | | | 21.3 % | | | | | | |
| Frequently | | 6/47 | | | 12.8 % | | | | | | |
| Most frequently | | 15/47 | | | 31.9 % | | | | | | |
| *Others* | |  | | |  | | | | | | |
| Never | | 8/10 | | | 80.0 % | | | | | | |
| Very rarely | | 1/10 | | | 10.0 % | | | | | | |
| Rarely | | 1/10 | | | 10.0 % | | | | | | |
| Occasionally | | 0/10 | | | 0.0 % | | | | | | |
| Frequently | | 0/10 | | | 0.0 % | | | | | | |
| Most frequently | | 0/10 | | | 0.0 % | | | | | | |
| **21 Common dose concept for SBRT of spine metastases** | | | |  |  | | | | | | |
| *Single-dose RT* | |  | | |  | | | | | | |
| Never | | 31/47 | | | 66.0 % | | | | | | |
| Very rarely | | 6/47 | | | 12.8 % | | | | | | |
| Rarely | | 5/47 | | | 10.6 % | | | | | | |
| Occasionally | | 4/47 | | | 8.5 % | | | | | | |
| Frequently | | 1/47 | | | 2.1 % | | | | | | |
| Most frequently | | 0/47 | | | 0.0 % | | | | | | |
| *hypofractionated SBRT daily* | |  | | |  | | | | | | |
| Never | | 2/42 | | | 4.8 % | | | | | | |
| Very rarely | | 4/42 | | | 9.5 % | | | | | | |
| Rarely | | 5/42 | | | 11.9 % | | | | | | |
| Occasionally | | 5/42 | | | 11.9 % | | | | | | |
| Frequently | | 20/42 | | | 47.6 % | | | | | | |
| Most frequently | | 6/42 | | | 14.3 % | | | | | | |
| *hypofractionated SBRT every other day* | |  | | |  | | | | | | |
| Never | | 16/40 | | | 40.0 % | | | | | | |
| Very rarely | | 1/40 | | | 2.5 % | | | | | | |
| Rarely | | 6/40 | | | 15.0 % | | | | | | |
| Occasionally | | 8/40 | | | 20.0 % | | | | | | |
| Frequently | | 7/40 | | | 17.5 % | | | | | | |
| Most frequently | | 2/40 | | | 5.0 % | | | | | | |
| *SIB* | |  | | |  | | | | | | |
| Never | | 8/42 | | | 19.1 % | | | | | | |
| Very rarely | | 6/42 | | | 14.3 % | | | | | | |
| Rarely | | 5/42 | | | 11.9 % | | | | | | |
| Occasionally | | 6/42 | | | 14.3 % | | | | | | |
| Frequently | | 13/42 | | | 31.0 % | | | | | | |
| Most frequently | | 4/42 | | | 9.5 % | | | | | | |
| **22 Common dose concept for SBRT of non-spine metastases** | | |  | |  | | | | | | |
| *Single-dose RT* | |  | | |  | | | | | | |
| Never | | 26/42 | | | 61.9 % | | | | | | |
| Very rarely | | 4/42 | | | 9.5 % | | | | | | |
| Rarely | | 5/42 | | | 11.9 % | | | | | | |
| Occasionally | | 6/42 | | | 14.3 % | | | | | | |
| Frequently | | 1/42 | | | 2.4 % | | | | | | |
| Most frequently | | 0/42 | | | 0.0 % | | | | | | |
| *hypofractionated SBRT daily* | |  | | |  | | | | | | |
| Never | | 4/40 | | | 10.0 % | | | | | | |
| Very rarely | | 4/40 | | | 10.0 % | | | | | | |
| Rarely | | 8/40 | | | 20.0 % | | | | | | |
| Occasionally | | 5/40 | | | 12.5 % | | | | | | |
| Frequently | | 14/40 | | | 35.0 % | | | | | | |
| Most frequently | | 5/40 | | | 12.5 % | | | | | | |
| *hypofractionated SBRT every other day* | |  | | |  | | | | | | |
| Never | | 15/39 | | | 38.5 % | | | | | | |
| Very rarely | | 0/39 | | | 0.0 % | | | | | | |
| Rarely | | 4/39 | | | 10.3 % | | | | | | |
| Occasionally | | 11/39 | | | 28.2 % | | | | | | |
| Frequently | | 7/39 | | | 17.9 % | | | | | | |
| Most frequently | | 2/39 | | | 5.1 % | | | | | | |
| *SIB* | |  | | |  | | | | | | |
| Never | | 11/39 | | | 28.2 % | | | | | | |
| Very rarely | | 7/39 | | | 17.9 % | | | | | | |
| Rarely | | 7/39 | | | 17.9 % | | | | | | |
| Occasionally | | 4/39 | | | 10.3 % | | | | | | |
| Frequently | | 7/39 | | | 17.9 % | | | | | | |
| Most frequently | | 3/39 | | | 7.7 % | | | | | | |
| **23 In-loco stereotactic re-irradiation of BoM** | |  | | |  | | | | | | |
| yes – after conventional pre-irradiation | | 45/50 | | | 90.0 % | | | | | | |
| yes - after stereotactic pre-irradiation | | 14/50 | | | 28.0 % | | | | | | |
| No | | 4/50 | | | 8.0 % | | | | | | |
| *If yes, at the earliest after which time interval?* | |  | | |  | | | | | | |
| < 6 months | | 6/33 | | | 18.2 % | | | | | | |
| 6 months | | 20/33 | | | 60.6 % | | | | | | |
| 6-12 months | | 3/33 | | | 9.1 % | | | | | | |
| > 12 months | | 4/33 | | | 12.1 % | | | | | | |
| **D Execution of SRT of Bone Metastases** | | **n** | | | **%** | | | | | | |
| **24 Treatment machines used for bone SBRT** | |  | | |  | | | | | | |
| *Conventional LINAC* | |  | | |  | | | | | | |
| Never | | 12/41 | | | 29.3 % | | | | | | |
| Very rarely | | 0/41 | | | 0.0 % | | | | | | |
| Rarely | | 1/41 | | | 2.4 % | | | | | | |
| Occasionally | | 3/41 | | | 7.3 % | | | | | | |
| Frequently | | 6/41 | | | 14.6 % | | | | | | |
| Most frequently | | 19/41 | | | 46.3 % | | | | | | |
| *Conventional LINAC with external X-ray verification for SRT* | |  | | |  | | | | | | |
| Never | | 18/43 | | | 41.9 % | | | | | | |
| Very rarely | | 0/43 | | | 0.0 % | | | | | | |
| Rarely | | 0/43 | | | 0.0 % | | | | | | |
| Occasionally | | 3/43 | | | 6.7 % | | | | | | |
| Frequently | | 6/43 | | | 14.0 % | | | | | | |
| Most frequently | | 16/43 | | | 37.2 % | | | | | | |
| *Dedicated LINAC with external X-ray verification for SRT* | |  | | |  | | | | | | |
| Never | | 21/39 | | | 53.9 % | | | | | | |
| Very rarely | | 0/39 | | | 0.0 % | | | | | | |
| Rarely | | 0/39 | | | 0.0 % | | | | | | |
| Occasionally | | 2/39 | | | 5.1 % | | | | | | |
| Frequently | | 5/39 | | | 12.8 % | | | | | | |
| Most frequently | | 11/39 | | | 28.2 % | | | | | | |
| *Helical radiotherapy* | |  | | |  | | | | | | |
| Never | | 27/36 | | | 75.0 % | | | | | | |
| Very rarely | | 2/36 | | | 5.6 % | | | | | | |
| Rarely | | 1/36 | | | 2.8 % | | | | | | |
| Occasionally | | 2/36 | | | 5.6 % | | | | | | |
| Frequently | | 2/36 | | | 5.6 % | | | | | | |
| Most frequently | | 2/36 | | | 5.6 % | | | | | | |
| *Robot-assisted radiosurgery* | |  | | |  | | | | | | |
| Never | | 28/37 | | | 75.7 % | | | | | | |
| Very rarely | | 1/37 | | | 2.7 % | | | | | | |
| Rarely | | 0/37 | | | 0.0 % | | | | | | |
| Occasionally | | 3/37 | | | 8.1 % | | | | | | |
| Frequently | | 0/37 | | | 0.0 % | | | | | | |
| Most frequently | | 5/37 | | | 13.5 % | | | | | | |
| *MR-LINAC* | |  | | |  | | | | | | |
| Never | | 32/35 | | | 91.4 % | | | | | | |
| Very rarely | | 1/35 | | | 2.9 % | | | | | | |
| Rarely | | 1/35 | | | 2.9 % | | | | | | |
| Occasionally | | 1/35 | | | 2.9 % | | | | | | |
| Frequently | | 0/35 | | | 0.0 % | | | | | | |
| Most frequently | | 0/35 | | | 0.0 % | | | | | | |
| *Proton therapy* | |  | | |  | | | | | | |
| Never | | 35/35 | | | 100.0 % | | | | | | |
| Very rarely | | 0/35 | | | 0.0 % | | | | | | |
| Rarely | | 0/35 | | | 0.0 % | | | | | | |
| Occasionally | | 0/35 | | | 0.0 % | | | | | | |
| Frequently | | 0/35 | | | 0.0 % | | | | | | |
| Most frequently | | 0/35 | | | 0.0 % | | | | | | |
| *Others* | |  | | |  | | | | | | |
| Never | | 25/27 | | | 92.6 % | | | | | | |
| Very rarely | | 1/27 | | | 3.7 % | | | | | | |
| Rarely | | 0/27 | | | 0.0 % | | | | | | |
| Occasionally | | 1/27 | | | 3.7 % | | | | | | |
| Frequently | | 0/27 | | | 0.0 % | | | | | | |
| Most frequently | | 0/27 | | | 0.0 % | | | | | | |
| **25 Verification systems used for bone SBRT** | |  | | |  | | | | | | |
| *Pretherapeutic 2D-imaging (including stereoscopic imaging)* | |  | | |  | | | | | | |
| Never | | 21/30 | | | 70.0 % | | | | | | |
| Very rarely | | 1/30 | | | 3.3 % | | | | | | |
| Rarely | | 3/30 | | | 10.0 % | | | | | | |
| Occasionally | | 2/30 | | | 6.7 % | | | | | | |
| Frequently | | 2/30 | | | 6.7 % | | | | | | |
| Most frequently | | 1/30 | | | 3.3 % | | | | | | |
| *Dynamic 2D-X-ray-imaging (e. g. intrafractional)* | |  | | |  | | | | | | |
| Never | | 21/32 | | | 65.6 % | | | | | | |
| Very rarely | | 0/32 | | | 0.0 % | | | | | | |
| Rarely | | 3/32 | | | 9.4 % | | | | | | |
| Occasionally | | 3/32 | | | 9.4 % | | | | | | |
| Frequently | | 3/32 | | | 9.4 % | | | | | | |
| Most frequently | | 2/32 | | | 6.3 % | | | | | | |
| *Pretherapeutic 3D-imaging (conebeam-CT)* | |  | | |  | | | | | | |
| Never | | 3/46 | | | 6.5 % | | | | | | |
| Very rarely | | 0/46 | | | 0.0 % | | | | | | |
| Rarely | | 0/46 | | | 0.0 % | | | | | | |
| Occasionally | | 0/46 | | | 0.0 % | | | | | | |
| Frequently | | 4/46 | | | 8.7 % | | | | | | |
| Most frequently | | 39/46 | | | 84.8 % | | | | | | |
| *2D-imaging after correction/table adjustment (including stereoscopic imaging)* | |  | | |  | | | | | | |
| Never | | 19/34 | | | 55.9 % | | | | | | |
| Very rarely | | 3/34 | | | 8.8 % | | | | | | |
| Rarely | | 1/34 | | | 2.9 % | | | | | | |
| Occasionally | | 1/34 | | | 2.9 % | | | | | | |
| Frequently | | 4/34 | | | 11.8 % | | | | | | |
| Most frequently | | 6/34 | | | 17.7 % | | | | | | |
| *3D-imaging after correction/table adjustment* | |  | | |  | | | | | | |
| Never | | 8/40 | | | 20.0 % | | | | | | |
| Very rarely | | 4/40 | | | 10.0 % | | | | | | |
| Rarely | | 1/40 | | | 2.5 % | | | | | | |
| Occasionally | | 4/40 | | | 10.0 % | | | | | | |
| Frequently | | 6/40 | | | 15.0 % | | | | | | |
| Most frequently | | 17/40 | | | 42.5 % | | | | | | |
| *Surface imaging* | |  | | |  | | | | | | |
| Never | | 14/37 | | | 37.8 % | | | | | | |
| Very rarely | | 2/37 | | | 5.4 % | | | | | | |
| Rarely | | 0/37 | | | 0.0 % | | | | | | |
| Occasionally | | 3/37 | | | 8.1 % | | | | | | |
| Frequently | | 8/37 | | | 21.6 % | | | | | | |
| Most frequently | | 10/37 | | | 27.0 % | | | | | | |
| *Others* | |  | | |  | | | | | | |
| Never | | 11/16 | | | 68.8 % | | | | | | |
| Very rarely | | 2/16 | | | 12.5 % | | | | | | |
| Rarely | | 0/16 | | | 0.0 % | | | | | | |
| Occasionally | | 1/16 | | | 6.3 % | | | | | | |
| Frequently | | 0/16 | | | 0.0 % | | | | | | |
| Most frequently | | 2/16 | | | 12.5 % | | | | | | |
| **E Follow-up after SRT of Bone Metastases** | | **n** | | | **%** | | | | | | |
| **26 Standard interval for clinical follow-up after bone SBRT** | |  | | |  | | | | | | |
| Quarterly | | 24/49 | | | 49.0 % | | | | | | |
| semi-annually | | 4/49 | | | 8.1 % | | | | | | |
| Annually | | 2/49 | | | 4.1 % | | | | | | |
| follow-up is organized by the primary treating specialist (e.g. oncologist) | | 8/49 | | | 16.3 % | | | | | | |
| others | | 11/49 | | | 21.5 % | | | | | | |
| **27 Standard interval for imaging follow-up after bone SBRT** | |  | | |  | | | | | | |
| not performed | | 8/49 | | | 16.3 % | | | | | | |
| only once | | 6/49 | | | 12.2 % | | | | | | |
| Quarterly | | 12/49 | | | 24.5 % | | | | | | |
| semi-annually | | 1/49 | | | 2.0 % | | | | | | |
| Annually | | 0/49 | | | 0.0 % | | | | | | |
| follow-up is organized by the primary treating specialist (e.g. oncologist) | | 14/49 | | | 28.6 % | | | | | | |
| others | | 8/49 | | | 16.3 % | | | | | | |
| **28 Use of structured/standardized instruments for evaluation** | |  | | |  | | | | | | |
| none | | 23/49 | | | 46.9 % | | | | | | |
| VAS/NRS | | 18/49 | | | 36.7 % | | | | | | |
| SINS-Score | | 12/49 | | | 24.5 % | | | | | | |
| Bilsky-Score | | 10/49 | | | 20.4 % | | | | | | |
| QoL-surveys | | 4/49 | | | 8.2 % | | | | | | |
| other | | 2/49 | | | 4.1 % | | | | | | |
